# Thrombophilia Associated with Anti-DFS70 Autoantibodies

**DOI:** 10.1371/journal.pone.0138671

**Published:** 2015-09-23

**Authors:** Julien Marlet, Annick Ankri, Jean-Luc Charuel, Pascale Ghillani-Dalbin, Amélie Perret, Isabelle Martin-Toutain, Julien Haroche, Zahir Amoura, Lucile Musset, Makoto Miyara

**Affiliations:** 1 Département d’Immunologie, Hôpital Pitié-Salpêtrière (AP-HP), Paris, France; 2 Laboratoire d’Hématologie, Hôpital Pitié-Salpêtrière (AP-HP), Paris, France; 3 INSERM U1135, Centre d’Immunologie et des Maladies Infectieuses (CIMI), Paris, France; 4 Service de Médecine Interne 2, institut E3M, Centre de référence national du lupus et du syndrome des antiphospholipides, Hôpital Pitié-Salpêtrière (AP-HP), Paris, France; University Hospital Basel, SWITZERLAND

## Abstract

**Context:**

Anti-DFS70 antibodies are the most frequent antinuclear antibodies (ANA) found in healthy individuals. We assessed the clinical significance of the presence of anti-DFS70 antibodies.

**Methods:**

We defined a group of patients (n = 421) with anti-DFS70 antibodies and a group of patients (n = 63) with a history of idiopathic arterial and/or venous thrombotic disease and/or obstetric complication (i.e. ≥3 miscarriages, fetal death or premature birth with eclampsia). Anti-DFS70 antibodies prevalence was also assessed in a cohort of 300 healthy blood donors.

**Results:**

The prevalence of thrombotic disease and/or obstetric complication in the 421 patients with anti-DFS70 antibodies was 13.1% (n = 55) and the prevalence of connective tissue disease was 19% (n = 80). Among the 63 patients with a history of thrombosis and/or obstetric complications, 7 (11.1%) had anti-DFS70 antibodies and among the latter, 5 had no common thrombophilic factor. In contrast, the prevalence of anti-DFS70 antibodies was of 3.0% (9 out of 300) in healthy donors. Finally, the Activated Partial Thromboplastin Time (aPTT) ratio of patients with a history of thrombosis and anti-DFS70 antibodies was lower than the aPTT ratio of other patients, suggesting that thrombotic patients with anti-DFS70 antibodies may have a hypercoagulable state.

**Conclusion:**

We described here for the first time an immune procoagulant state involving anti-DFS70 antibodies.

## Introduction

**Fig 1:**
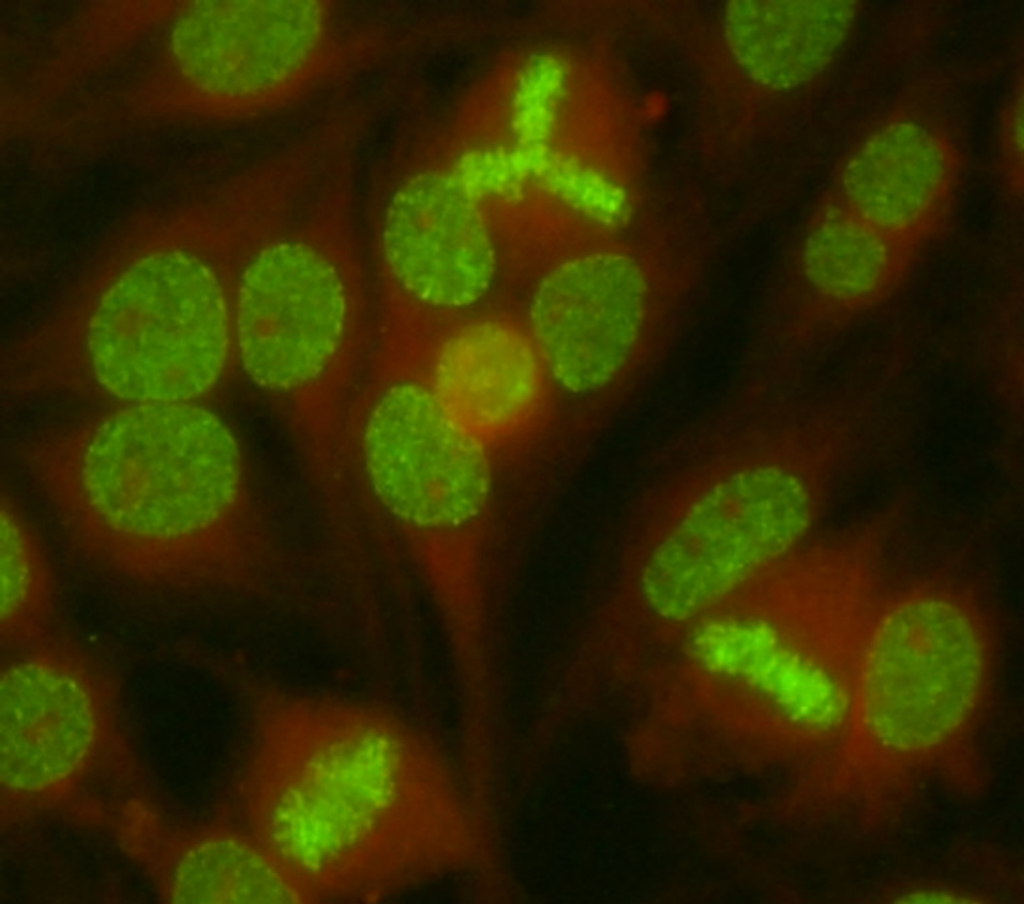
Dense Fine Speckled nuclear pattern on HEp-2000® cells (titer 1:1280). Indirect Immunofluorescence on Hep2000® cells with a fluorescein-conjugated secondary antibody (Immunoconcept) of the serum of a patient with anti-DFS70 antibodies. Picture was acquired using a LEICA/DM-LB2 microscope (x400 magnification) at 20°C, using camera DFC 300FX and acquisition software IM500 (Leica). Anti-DFS70 antibodies titer was determined by testing successive two-fold dilutions of the serum from 1/80 to 1/1280. Samples were classified as positive if well-defined immunofluorescence patterns were identified at 1/160 dilution.

The search for antinuclear antibodies (ANA) by indirect immunofluorescence (IIF) on HEp-2 cells is routinely performed as the first step for the biological diagnosis of systemic autoimmune diseases [1–3]. Anti-DFS70 antibodies are a type of ANA defined by a nuclear dense fine speckled (DFS) IIF pattern, first described in 1994 (Fig 1) [4]. Anti-DFS70 antibodies recognize the Lens Epithelium Derived Growth Factor antigen (LEDGF), a nuclear protein involved in DNA remodeling and later identified as the transcription coactivator p75 [5]. Trained immunologist can easily distinguish this IIF pattern from the ones commonly observed in connective tissue diseases (CTD) and we have recently shown that the DFS IIF pattern indeed corresponded to the presence of anti-LEDGF antibodies detected by specific assays [6]. Descriptions of the clinical features of patients with anti-DFS70 antibodies have shown that they were associated with interstitial cystitis [4], atopic dermatitis [7], alopecia areata [8], cataract and Vogt-Koyanagi-Harada disease [9]. It has also been reported that anti-DFS70 antibodies (at titers ≥ 1:160) were the most frequent type of ANA found in healthy individuals with a prevalence of 6% [10, 11]. A more recent study indicated that anti-DFS70 antibodies were observed at a lower frequency in healthy donors (3%) while they were absent in CTD [12]. We recently showed that anti-DFS70 antibodies could be detected during CTD while 12% of patients with anti-DFS70 antibodies had ongoing CTD [6]. Because those studies were conducted on a limited number of individuals, we conducted a retrospective study on a larger number of consecutive patients tested positive for anti-DFS70 antibodies (n = 421). Here, we show that a significant proportions of patients harboring anti-DFS70 antibodies unexpectedly presented with a history of thrombosis (arterial or venous) or obstetric complications including miscarriages, fetal death and premature birth with eclampsia that were not explained by the presence of common thrombophilic factors [13].

## Materials and Methods

### Patient inclusion

The first group of consecutive patients was selected among all patients (n = 16 754) undergoing routine antinuclear antibodies testing (ANA) at the Pitié-Salpêtrière hospital (Paris, France) between the 1st of June 2011 and ended on the 31st of July 2013. All patients (n = 421) with ANA and a DFS pattern at titer higher or equal to 1:160 were included [14]. ANA testing was performed to diagnose CTD in internal medicine or to investigate a history of thrombosis in hematology. One hundred patients, included between the 7^th^ of December 2011 and the 25^th^ of April 2012 have been previously described for the prevalence of CTD but not for other pathological conditions [6]. We defined a second group of patients (n = 63) followed-up in hematology department at the Pitié-Salpêtrière hospital (Paris, France) who had a history of confirmed idiopathic arterial thrombosis (i.e. myocardial infarction and/or ischemic stroke) and/or venous thromboembolism (VTE) including deep vein thrombosis (DVT) and pulmonary embolism (PE) and/or obstetric complications that included ≥3 miscarriages before the 10^th^ week of gestation, death of a morphologically normal fetus after the 10^th^ week of gestation or premature birth with eclampsia before the 34^th^ week of gestation. Patient inclusion started on the 1^st^ of January 2013 and ended on the 31^st^ of December 2013. Patients in both groups were evaluated with thrombophilia testing that included the search for thrombophilic factors [13], i.e. factor V G1691A (Leiden), prothrombin G20210A and MTHFR C677T mutations [15–19], antithrombin (AT), protein C (PC) and protein S (PS) deficiencies [20, 21], antiphospholipid antibodies (Lupus anticoagulant, Anticardiolipin and anti-β2 glycoprotein 1 antibodies of IgG and IgM isotypes), in addition to ANA testing. The clinical history of all patients was retrospectively analyzed by clinical chart review of medical records and diagnoses were established according to disease criteria for respective diseases. CTD included systemic lupus erythematosus (SLE), rheumatoid arthritis (RA), Sjögren syndrome (SjS), systemic sclerosis (SS), polymyositis (PM) or mixed connective tissue disease (MCTD) [22–24]. Similarly, thrombotic disease was defined as either a history of confirmed arterial thrombosis and/or VTE and/or obstetric complications (as defined above [25]. Data were anonymously used in accordance with the latest version of the Helsinki Declaration of human research ethics. Collection of patient samples was carried out according to local ethics committee regulations and ethical approval was obtained from the “CPP—Ile de France- VI” at the Pitié-Salpêtrière Hospital. No consent was needed from any patients involved in this study. Anti-DFS70 antibodies prevalence was also assessed in a cohort of 300 anonymous healthy blood donors that did not experienced thrombotic event or obstetric event (Etablissement français du sang, Paris, France).

### Activated Partial Thromboplastin Time (aPTT)

Venous blood samples were collected into 5 ml tubes containing one-tenth volume 0.109 M trisodium citrate into Vacutainer tubes (Becton Dickinson) using a minimal stasis. Platelet-poor plasma (PPP) was prepared by centrifugation at room temperature at 3000 g for 15 minutes, and was analyzed within 3 h of collection. The aPTT (TriniCLOT™ Automated aPTT, Tcoag) was determined on a STA-R Evolution instrument (Stago, Asnières, France) according to the manufacturer recommendations. Results are expressed as ratio of patient-to-pooled normal plasma. The local cut-off ratio determined for aPTT was determined using PPP from 50 apparently healthy volunteers.

### Indirect immunofluorescence (IIF)

IIF was performed using HEp-2000® cells and secondary anti-human IgG supplied by Immunoconcept (Sacramento, USA), according to the manufacturer’s instructions. The screening dilution was 1:80. Readings were performed by technicians and confirmed by a specialized biologist on a LEICA DM LB2 at the objective x40. Anti-DFS IIF pattern was characterized by dense fine speckles distributed throughout the nucleus.

### Statistical analysis

Data were analyzed using two-tailed Fisher exact test, z-test, non parametric Mann-Whitney U-test and Chi-2 test with GraphPad Prism 5 to analyze the differences between groups. For all statistical tests, p-value < 0.05 was considered significant.

## Results

### Low prevalence of connective tissue diseases in patients with anti-DFS70 antibodies

Among the 16754 single patients tested for ANA, 421 patients (2.5%) had anti-DFS70 antibodies (Table 1); 83% were females (n = 348, female/male sex ratio 4.8), 52 patients had SLE (12.4%), 9, RA (2.1%), 15, primary Sjögren syndrome (3.6%), 2, inflammatory myositis (0.5%) and 2 had mixed connective tissue diseases (0.5%). Most of patients consulted in internal medicine (n = 227, 54%) with a low prevalence of CTD (n = 80, 19.0%). Among the patients without CTD, 15 patients had multiple sclerosis (3.6%), 16 had thyroiditis (3.8%) and 2 had atopic dermatitis (0.5%) but none had alopecia areata [8], interstitial cystitis [4], or Vogt-Koyanagi-Harada disease [10].

**Table 1:** Description of patients with anti-DFS70 antibodies.

	History of thrombosis and/or obstetric events		
	Yes (n = 55; 13.1%)	No (n = 366; 86.9%)	All patients (n = 421)
CTD	13 (24%)	69 (19%)	82 (20%)
Sex ratio F:M	4.0 (44:11)	4.9 (304:62)	4.8 (348:73)
Age (mean±SD; median and range)	43 (±16); 41 (16–82)	44 (±16); 42 (13–87)	44 (±16); 42 (13–87)
*Referring physicians*			
Internal medicine	39 (71%)*	188 (51%)*	227 (54%)
Hematology	13 (24%)*	10 (3%)*	23 (6%)
Other	3 (6%)*	168 (46%)*	171 (41%)

*Statistically significant differences (p-value < 0.05)

### A subset of patients with anti-DFS70 antibodies has a history of thrombosis or obstetric complications

Among the 421 patients with anti-DFS70 antibodies, thrombosis and/or obstetric complications (defined above) occurred at an unexpectedly high prevalence (n = 55, 13.1%). Respective prevalences of VTE and arterial thrombosis were 6.2% (n = 26) and 3.1% (n = 13) while the prevalence of obstetric complications was 5.8% in female patients (n = 20). Clinical and biological features for those 55 patients are detailed in S1 Table. Four patients had both VTE and obstetric complications; one had arterial and VTE while another had arterial thrombosis and obstetric complications. No patients combined arterial and VTE and obstetric complications. Among the 55 patients with anti-DFS70 antibodies and a history of thrombosis and/or obstetric complications, 121 events (either thrombotic or obstetric) were observed with a mean of 2.2 (±1.5) events per patient and a median number of events of 2 (Range: 1–7). Among male patients (n = 11), 17 thrombotic events occurred (1.55±0.93 events per patient), while among female patients (n = 44), 104 thrombotic or obstetric events occurred (2.36±1.53 events per patient). Among those 104 events in female patients, 53 were obstetric complications (1.20±1.76 events per patient). Therefore, the most prevalent events were obstetric complications (44%, n = 53) and VTE (40%, n = 49) while arterial thrombosis represented 16% (n = 19) of events. The nature of the first event observed in patients was VTE in 44% of patients (n = 24), obstetric complications in 39% of women (n = 17) and arterial thrombosis in 25% of patients (n = 14), with a mean age of 32 (±13) for VTE, 32 (±6) for obstetric complications and 43 (±12) for arterial thrombosis. A majority of those 55 patients (n = 39, 71%) had neither CTD nor APS. Because of the retrospective status of the study, only 34 of those 55 patients were tested for thrombophilia; among these 34 patients, 20 (59%) did not have any known thrombophilic factor. We compared the patients with a history of thrombosis and/or obstetric complications with the patients without thrombotic history and we did not found any significant differences in term of age, gender, CTD prevalence (Table 1) and anti-DFS70 antibodies titers (S1 Fig).

### A significant proportion of patients with unexplained thrombophilia have anti-DFS70 antibodies

Because we have shown above that patients with anti-DFS70 antibodies may be more prone to develop thrombosis and/or obstetric complications, we investigated whether anti-DFS70 antibodies were prevalent in patients suspected with thrombophilia. We thus constituted a thrombosis group that included patients consulting for the exploration of thrombophilia (i.e. either arterial or venous single or recurrent idiopathic thrombosis and/or unexplained obstetric complications as defined above). Therefore, this thrombosis group included 63 patients, whose clinical and biological features are summarized in Table 2. The prevalence of anti-DFS70 antibodies (n = 7, 11.1%) in the 63 thrombophilic patients cohort was higher, although not statistically significantly, than the prevalence of factor V Leiden (9%, n = 6), G20210A polymorphism of prothrombin gene (8%, n = 5), AT and PS deficiencies (3%, n = 2 for each) and PC deficiency (n = 0) but lower than the prevalence of homozygote C677T polymorphism of MTHFR gene (13%, n = 9) and antiphospholipid antibodies (13.4%, n = 9). Thirty-eight patients had no known thrombophilic factor. Among the latter, five patients harbored anti-DFS70 antibodies. Therefore, patients with anti-DFS70 antibodies represented 13% of the 38 patients without any known thrombophilic factor. In comparison, the prevalence of anti-DFS70 antibodies in 300 healthy blood donors without thrombotic or obstetric events was lower (3.0%, n = 9). The odds ratio for thrombotic events in the presence of anti-DFS70 antibodies was therefore of 4.04 (CI_95%_: 1.44–11.3). Altogether, our findings indicate that a previously undescribed subset of patients with unexplained thrombosis and/or obstetric complications harbors anti-DFS70 antibodies.

**Table 2:** Description of patients with a history of thrombotic and/or obstetric events.

History of thrombotic and/or obstetric events among:	Patients with anti-DFS70 antibodies (n = 55)	Patients with thrombophilia (n = 63)	p-value
*Nature of events*			
Arterial thrombosis	13 (24%)	6 (10%)	<0.05*
Venous thrombosis	26 (47%)	55 (87%)	<0.001*
Obstetric syndrome	20 (46% of women)	6 (13% of women)	<0.001*
*Risk factors of thrombosis*			
Anti-DFS70 antibodies	55 (100%)	7 (11%)	<0.001*
Thrombophilic factors	14 (41%)†	24 (38%)	0.7
*Clinical context*			
Sex ratio F:M	4.0 (44:11)	2.2 (46:21)	0.40
Age (mean±SD); median and range)	43 (±16); 41 (16–82)	50 (±16); 50 (21–82)	0.04*
CTD	13 (24%)	None	<0.001*
APS	8 (15%)	9 (13%)	1.0
*Referring physicians*			
Hematology	13 (24%)*	63 (100%)*	<0.001*

*Statistically significant differences (p-value<0.05). CTD, Connective Tissue Disease, APS, Antiphospholipid antibodies syndrome. Thrombophilic factors included factor V Leiden, G20210A polymorphism of prothrombin gene and C677T polymorphism of MTHFR gene, antiphospholipid antibodies (Lupus anticoagulant, IgG, IgM), antithrombin III, protein C and protein S deficiencies.

^†^ Due to the retrospective status of the study, only 34 of the 55 patients were tested for thrombophilia.

### Patients with anti-DFS70 antibodies have a hypercoagulable state

**Fig 2:**
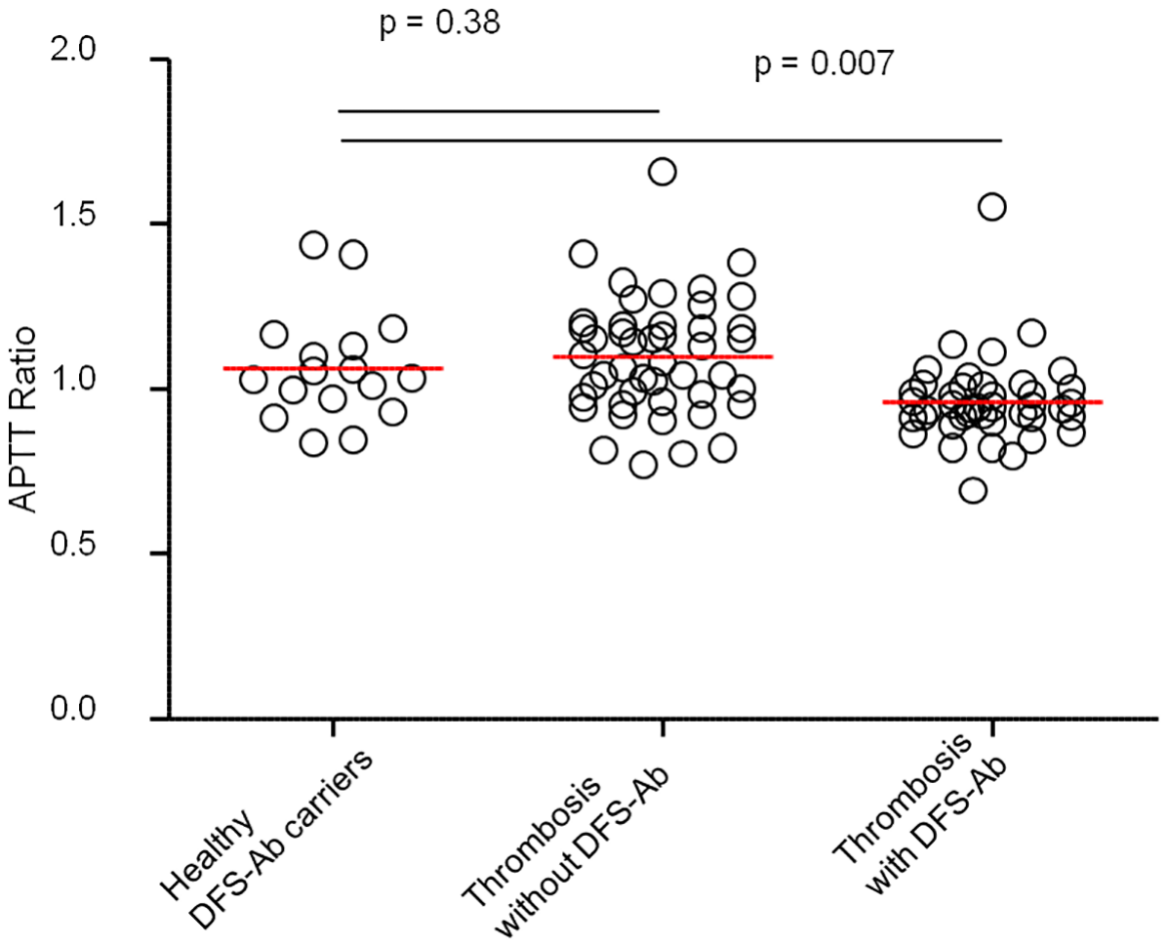
Hypercoagulable state in thrombotic patients harboring anti-DFS70 antibodies. APTT ratio of healthy individuals with anti-DFS70 antibodies (DFS-Ab, n = 17), patients with thrombophilia without anti-DFS70 antibodies (n = 46) and patients with thrombosis and anti-DFS70 antibodies (n = 42) are shown. Red horizontal lines represent mean values. Comparisons were made using non parametric Mann-Whitney U-test. P-values under 0.05 were considered significant.

Finally we assessed whether the prevalence of thrombosis in patients carrying anti-DFS70 antibodies could be mechanistically explained. Hypercoagulable state can be detected with routinely performed aPTT which investigates the activity of proacoagulant factors like IX, XI, II and fibrinogen, as shortened aPTT has been shown to be associated with VTE [26]. We thus retrospectively compared the aPTT measured in the absence of any therapeutic anticoagulant treatments of (1) patients with anti-DFS70 antibodies that had experienced thrombosis (n = 42) with those of (2) healthy anti-DFS70 antibodies carriers (n = 17) and of (3) patients that experienced thrombosis without anti-DFS70 antibodies (n = 46). As shown in Fig 2, healthy anti-DFS70 antibodies carriers had a mean+/-SD ratio of 1.062+/-0.17 while patients with thrombosis without anti-DFS70 antibodies had a mean+/-SD ratio of 1.10 +/-0.18. Patients that experienced thrombosis in the presence of anti-DFS70 antibodies had a mean+/-SD ratio of 0.95 +/-0.119. The latter patients ratio was significantly lower than those of healthy anti-DFS70 antibodies carriers (p = 0.007) while those of healthy anti-DFS70 antibodies carriers was not significantly different from those of patients with thrombosis without anti-DFS70 antibodies (p = 0.34). Of note, some patients with a history of thrombosis and anti-DFS70 antibodies had ratios as low as 0.69. Finally, when carefully analyzing the healthy anti-DFS70 antibodies carriers, we could notice that some patients displayed low aPTT ratio, close to those observed in thrombotic patients with anti-DFS70 antibodies. We can therefore hypothesize that those patients might be at risk of thrombotic events. Therefore, we conclude that thrombotic patients with anti-DFS70 antibodies might be in a state of hypercoagulability.

## Discussion

In this study, we constituted the largest cohort, to our knowledge, of patients with anti-DFS70 antibodies (n = 421). While we could confirm that CTD were not absent but with a low prevalence in patients with anti-DFS70 antibodies, we did not observed any specific association between anti-DFS70 antibodies and alopecia areata [8], interstitial cystitis [4], or Vogt-Koyanagi-Harada disease or atopic dermatitis [7, 10]. The commonly accepted definition for significant positivity for ANA is set to titers superior or equal to 1:160 [14]. The prevalence of anti-DFS70 antibodies has been previously reported in a large cohort of healthy individuals (n = 597) at 11% with a threshold of 1:40 [11]. Careful analysis of presented data in this study indicates that the prevalence of anti-DFS70 antibodies at significant titers (≥ 1:160) is rather of 5.8%. A more recent study conducted on a higher number of healthy individuals (n = 918) indicated that anti-DFS70 antibodies were present at significant titers with a lower prevalence (3.15%) [12]. We therefore assessed the prevalence of anti-DFS70 antibodies locally in 300 healthy blood donors and determined that it was indeed close to the ones calculated using commonly accepted thresholds (n = 9; 3.0%). We unexpectedly observed that idiopathic thrombosis and/or obstetric complications occurred in 13.1% (n = 55 out of 421) of patients with anti-DFS70 antibodies. In particular, VTE occurred in 6.2% of patients. It has been reported that, in a cohort of 11084 volunteers, VTE occurred with a lower prevalence (2.9%) [27]. Furthermore, obstetric complications were more frequent in women with anti-DFS70 antibodies than in a cohort of pregnant women (5.7% vs. 1–5%) while arterial thrombosis frequency seemed to be similar to that observed in volunteers (3.1% vs. 4.4%) [27, 28]. To minimize bias related to the retrospective aspect of the study, we calculated the rate of samples positive for anti-DFS70 antibodies in patients undergoing ANA test (n = 16754) and found a 2.5% rate of positivity, which is close to the rate of positivity (3%) found in the general population and in our health donor control group. We therefore state that the probability of bias due to the restrospective status of the study and recruitment bias are minimal. We can therefore hypothesize that the risk of VTE and obstetric complications may be higher in patients harboring anti-DFS70 antibodies. Moreover, anti-DFS70 antibodies were more prevalent in our group of consecutive patients with a history of thrombophilia (11.1%, 7 patients out of 63) than in both published cohorts of healthy individuals (5.8%, 35 patients out of 597 and 3.15%, 29 out of 918 respectively) or our cohort of healthy donors (3.0%, 9 patients out of 300). The calculation of the odds ratios (1.9, CI_95%_: 0.8–4.6; 3.7, CI_95%_: 1.55–8.7 calculated respectively from both studies and 4.04, CI_95%_: 1.44–11.3 when compared to our cohort of healthy donors) also suggests that anti-DFS70 antibodies may be associated with a high risk of thrombosis and/or obstetric complications. We have previously demonstrated that the presence of DFS IIF pattern observed on HEp-2 cells was equivalent to the detection of anti-LEDGF antibodies using specific assays, indicating that the sole IIF method is sufficient to determine the presence of anti-DFS70/anti-LEDGF antibodies [6]. LEDGF was first described as a nuclear protein, named p75 [29]. LEDGF has been described as a secreted protein with growth-factor properties, also responsible for resistance to thermal and oxidative stresses [30]. The biological effects of LEDGF have been shown to be increased in the presence of heparin [31, 32]. The capacity of heparin to interact with LEDGF may indicate that the LEDGF/anti-DFS70 antibodies immune complex may play a role in the induction of thrombosis. Furthermore, we observed that aPTT ratio was particularly reduced in patients with thrombosis and anti-DFS70 antibodies, indicating that the latter patients may be in a hypercoagulable state [26]. Given the large number of patients included and the large number of patients described here, we can assume that the presence of anti-DFS70 antibodies (at titers >1:160) may constitute an immune-mediated prothrombotic state at high risk for thrombotic adverse complications as observed in antiphospholipid syndrome or in heparin induced thrombocytopenia [33–35]. It is widely accepted that autoantibodies are present years before the occurrence of the first clinical event of autoimmune diseases. For instance, it has been demonstrated that specific autoantibodies could be detected up to 10 years before the clinical onset of CTD, antiphospholipid syndrome, rheumatoid arthritis or type 1 diabetes [36–38]. Therefore, it is not surprising that, first, anti-DFS70 antibodies can be present in apparently healthy donors and, second, that not all patients with anti-DFS70 antibodies have developed thrombotic or obstetric events. Of note, some healthy anti-DFS70 antibodies carriers displayed low aPTT ratio close to those observed in thrombotic patients with anti-DFS70 antibodies. It remains to be determined whether those healthy carriers are at higher risk of thrombosis than those with a normal aPTT ratio. An attempt to determine which epitopes of LEDGF were recognized by anti-DFS70 antibodies indicated that anti-DFS70 antibodies from 93 individuals invariably recognized a single conformational epitope located in the C-terminus region of LEDGF [39]. We could also confirm that all patients with anti-DFS70 antibodies detected using IIF methods harbored antibodies recognizing this epitope [6]. Taken together, our results suggest that patients tested positive for anti-DFS70 antibodies might be at risk for immune-mediated thrombotic events. Given the restrospective status of this study, further prospective studies are needed to confirm our results and to determine whether anti-DFS70 antibodies indeed represent a risk factor for thrombosis. Such studies would determine whether the search for anti-DFS70 antibodies screening by ANA testing in IIF could be included in the etiologic diagnosis procedures for thrombosis and/or obstetric complications and whether patients with anti-DFS70 antibodies might benefit from a more stringent follow-up and/or preventive therapeutic strategies.

## Supporting Information

S1 Fig Anti-DFS70 antibodies titers in indirect immunofluorescence. Distribution of anti-DFS70 antibodies titers in patients with or without thrombosis and/or obstetric complications is shown.
(TIF)

S1 Table Description of anti-DFS+ patients with APS-like events. F, Female; M, Male; CTD, Connective tissue disease; SLE, Systemic Lupus Erythematosus; SjS, Sjögren Syndrom; RA, Rheumatoid arthritis; AT, Arterial thrombosis; VT, Venous thrombosis; OE, Obstetric event including miscarriage, preeclampsia or HELLP syndrome; OCP, Oral Contraceptive Pills containing estrogen; APL, Anti-phospholipid Antibodies; Htz, Heterozygous, Hmz, Homozygous; NA, Not available; *, Patients also included in the thrombosis group. Cardiovascular risk factors included age (>50 for men and >60 for women), history of myocardial infarction in first-degree relatives (<55 for men, <65 for women), tobacco use, type 2 diabetes, HDL-cholesterol <0.4g/L. †, miscarriages before the 10^th^ week of gestation; ‡, fetal death after the 10^th^ week of gestation; #, premature birth with eclampsia before the 34^th^ week of gestation
(DOC)

## References

[1] Agmon-Levin N, Damoiseaux J, Kallenberg C, Sack U, Witte T, Herold M, et al International recommendations for the assessment of autoantibodies to cellular antigens referred to as anti-nuclear antibodies. Annals of the rheumatic diseases. 2014;73(1):17–23. Epub 2013/10/16. 10.1136/annrheumdis-2013-203863 . 24126457

[2] Solomon DH, Kavanaugh AJ, Schur PH. Evidence-based guidelines for the use of immunologic tests: antinuclear antibody testing. Arthritis and rheumatism. 2002;47(4):434–44. Epub 2002/09/05. 10.1002/art.10561 . 12209492

[3] Agmon-Levin N, Shapira Y, Selmi C, Barzilai O, Ram M, Szyper-Kravitz M, et al A comprehensive evaluation of serum autoantibodies in primary biliary cirrhosis. Journal of autoimmunity. 2010;34(1):55–8. Epub 2009/11/10. 10.1016/j.jaut.2009.08.009 . 19897339

[4] Ochs RL, Stein TW Jr., Peebles CL, Gittes RF, Tan EM. Autoantibodies in interstitial cystitis. The Journal of urology. 1994;151(3):587–92. Epub 1994/03/01. . 8308964 10.1016/s0022-5347(17)35023-1

[5] Daugaard M, Baude A, Fugger K, Povlsen LK, Beck H, Sorensen CS, et al LEDGF (p75) promotes DNA-end resection and homologous recombination. Nature structural & molecular biology. 2012;19(8):803–10. Epub 2012/07/10. 10.1038/nsmb.2314 . 22773103

[6] Miyara M, Albesa R, Charuel JL, El Amri M, Fritzler MJ, Ghillani-Dalbin P, et al Clinical phenotypes of patients with anti-DFS70/LEDGF antibodies in a routine ANA referral cohort. Clinical & developmental immunology. 2013;2013:703759 Epub 2013/03/12. 10.1155/2013/703759 ; PubMed Central PMCID: PMCPmc3580898. 23476678 PMC3580898

[7] Ochs RL, Muro Y, Si Y, Ge H, Chan EK, Tan EM. Autoantibodies to DFS 70 kd/transcription coactivator p75 in atopic dermatitis and other conditions. The Journal of allergy and clinical immunology. 2000;105(6 Pt 1):1211–20. Epub 2000/06/16. . 10856157 10.1067/mai.2000.107039

[8] Okamoto M, Ogawa Y, Watanabe A, Sugiura K, Shimomura Y, Aoki N, et al Autoantibodies to DFS70/LEDGF are increased in alopecia areata patients. Journal of autoimmunity. 2004;23(3):257–66. Epub 2004/10/27. 10.1016/j.jaut.2004.07.004 . 15501396

[9] Shinohara T, Singh DP, Chylack LT Jr. Review: Age-related cataract: immunity and lens epithelium-derived growth factor (LEDGF). Journal of ocular pharmacology and therapeutics: the official journal of the Association for Ocular Pharmacology and Therapeutics. 2000;16(2):181–91. Epub 2000/05/10. . 10803429 10.1089/jop.2000.16.181

[10] Yamada K, Senju S, Shinohara T, Nakatsura T, Murata Y, Ishihara M, et al Humoral immune response directed against LEDGF in patients with VKH. Immunology letters. 2001;78(3):161–8. Epub 2001/10/02. . 11578690 10.1016/s0165-2478(01)00243-7

[11] Watanabe A, Kodera M, Sugiura K, Usuda T, Tan EM, Takasaki Y, et al Anti-DFS70 antibodies in 597 healthy hospital workers. Arthritis and rheumatism. 2004;50(3):892–900. Epub 2004/03/17. 10.1002/art.20096 . 15022332

[12] Mariz HA, Sato EI, Barbosa SH, Rodrigues SH, Dellavance A, Andrade LE. Pattern on the antinuclear antibody-HEp-2 test is a critical parameter for discriminating antinuclear antibody-positive healthy individuals and patients with autoimmune rheumatic diseases. Arthritis and rheumatism. 2011;63(1):191–200. Epub 2010/10/19. 10.1002/art.30084 . 20954189

[13] National Clinical Guideline C. National Institute for Health and Clinical Excellence: Guidance Venous Thromboembolic Diseases: The Management of Venous Thromboembolic Diseases and the Role of Thrombophilia Testing. London: Royal College of Physicians (UK)National Clinical Guideline Centre; 2012. 23638495

[14] Tan EM, Feltkamp TE, Smolen JS, Butcher B, Dawkins R, Fritzler MJ, et al Range of antinuclear antibodies in "healthy" individuals. Arthritis and rheumatism. 1997;40(9):1601–11. Epub 1997/10/27. . 9324014 10.1002/art.1780400909

[15] Arruda VR, von Zuben PM, Chiaparini LC, Annichino-Bizzacchi JM, Costa FF. The mutation Ala677—>Val in the methylene tetrahydrofolate reductase gene: a risk factor for arterial disease and venous thrombosis. Thrombosis and haemostasis. 1997;77(5):818–21. Epub 1997/05/01. . 9184384

[16] Martinelli I, Taioli E, Cetin I, Marinoni A, Gerosa S, Villa MV, et al Mutations in coagulation factors in women with unexplained late fetal loss. The New England journal of medicine. 2000;343(14):1015–8. Epub 2000/10/06. 10.1056/nejm200010053431405 . 11018168

[17] Poort SR, Rosendaal FR, Reitsma PH, Bertina RM. A common genetic variation in the 3'-untranslated region of the prothrombin gene is associated with elevated plasma prothrombin levels and an increase in venous thrombosis. Blood. 1996;88(10):3698–703. Epub 1996/11/15. . 8916933

[18] Ridker PM, Hennekens CH, Lindpaintner K, Stampfer MJ, Eisenberg PR, Miletich JP. Mutation in the gene coding for coagulation factor V and the risk of myocardial infarction, stroke, and venous thrombosis in apparently healthy men. The New England journal of medicine. 1995;332(14):912–7. Epub 1995/04/06. 10.1056/nejm199504063321403 . 7877648

[19] De Stefano V, Martinelli I, Mannucci PM, Paciaroni K, Chiusolo P, Casorelli I, et al The risk of recurrent deep venous thrombosis among heterozygous carriers of both factor V Leiden and the G20210A prothrombin mutation. The New England journal of medicine. 1999;341(11):801–6. Epub 1999/09/09. 10.1056/nejm199909093411104 . 10477778

[20] Kupferminc MJ, Eldor A, Steinman N, Many A, Bar-Am A, Jaffa A, et al Increased frequency of genetic thrombophilia in women with complications of pregnancy. The New England journal of medicine. 1999;340(1):9–13. Epub 1999/01/08. 10.1056/nejm199901073400102 . 9878639

[21] Preston FE, Rosendaal FR, Walker ID, Briet E, Berntorp E, Conard J, et al Increased fetal loss in women with heritable thrombophilia. Lancet. 1996;348(9032):913–6. Epub 1996/10/05. . 8843809 10.1016/s0140-6736(96)04125-6

[22] Mahler M, Fritzler MJ. Epitope specificity and significance in systemic autoimmune diseases. Annals of the New York Academy of Sciences. 2010;1183:267–87. Epub 2010/02/12. 10.1111/j.1749-6632.2009.05127.x . 20146721

[23] Stinton LM, Fritzler MJ. A clinical approach to autoantibody testing in systemic autoimmune rheumatic disorders. Autoimmunity reviews. 2007;7(1):77–84. Epub 2007/10/31. 10.1016/j.autrev.2007.08.003 . 17967730

[24] Goldblatt F, O'Neill SG. Clinical aspects of autoimmune rheumatic diseases. Lancet. 2013;382(9894):797–808. Epub 2013/09/03. 10.1016/s0140-6736(13)61499-3 . 23993190

[25] Miyakis S, Lockshin MD, Atsumi T, Branch DW, Brey RL, Cervera R, et al International consensus statement on an update of the classification criteria for definite antiphospholipid syndrome (APS). Journal of thrombosis and haemostasis: JTH. 2006;4(2):295–306. Epub 2006/01/20. 10.1111/j.1538-7836.2006.01753.x . 16420554

[26] Tripodi A, Chantarangkul V, Martinelli I, Bucciarelli P, Mannucci PM. A shortened activated partial thromboplastin time is associated with the risk of venous thromboembolism. Blood. 2004;104(12):3631–4. Epub 2004/08/07. 10.1182/blood-2004-03-1042 . 15297315

[27] Biino G, Portas L, Murgia F, Vaccargiu S, Parracciani D, Pirastu M, et al A population-based study of an Italian genetic isolate reveals that mean platelet volume is not a risk factor for thrombosis. Thrombosis research. 2012;129(4):e8–13. Epub 2011/12/06. 10.1016/j.thromres.2011.11.018 . 22137741

[28] Kujovich JL. Thrombophilia and pregnancy complications. American journal of obstetrics and gynecology. 2004;191(2):412–24. Epub 2004/09/03. 10.1016/j.ajog.2004.03.001 . 15343215

[29] Ge H, Si Y, Roeder RG. Isolation of cDNAs encoding novel transcription coactivators p52 and p75 reveals an alternate regulatory mechanism of transcriptional activation. The EMBO journal. 1998;17(22):6723–9. Epub 1998/11/21. 10.1093/emboj/17.22.6723 ; PubMed Central PMCID: PMCPmc1171017. 9822615 PMC1171017

[30] Singh DP, Ohguro N, Chylack LT Jr., Shinohara T. Lens epithelium-derived growth factor: increased resistance to thermal and oxidative stresses. Investigative ophthalmology & visual science. 1999;40(7):1444–51. Epub 1999/06/08. . 10359326

[31] Chen FF, Lin WH, Lin SC, Kuo JH, Chu HY, Huang WC, et al Significance of heparin binding to basic residues in homologous to the amino terminus of hepatoma-derived growth factor and related proteins. Glycobiology. 2012;22(5):649–61. Epub 2012/01/10. 10.1093/glycob/cwr191 . 22223757

[32] Fatma N, Singh DP, Shinohara T, Chylack LT Jr. Heparin's roles in stabilizing, potentiating, and transporting LEDGF into the nucleus. Investigative ophthalmology & visual science. 2000;41(9):2648–57. Epub 2000/08/11. . 10937578

[33] Levine JS, Branch DW, Rauch J. The antiphospholipid syndrome. The New England journal of medicine. 2002;346(10):752–63. Epub 2002/03/08. 10.1056/NEJMra002974 . 11882732

[34] Kelton JG, Warkentin TE. Heparin-induced thrombocytopenia: a historical perspective. Blood. 2008;112(7):2607–16. Epub 2008/09/24. 10.1182/blood-2008-02-078014 . 18809774

[35] Yoon JH, Jang IK. Heparin-induced thrombocytopenia in cardiovascular patients: pathophysiology, diagnosis, and treatment. Cardiology in review. 2011;19(3):143–53. Epub 2011/04/06. 10.1097/CRD.0b013e318211f7c0 . 21464642

[36] Rantapaa-Dahlqvist S, de Jong BA, Berglin E, Hallmans G, Wadell G, Stenlund H, et al Antibodies against cyclic citrullinated peptide and IgA rheumatoid factor predict the development of rheumatoid arthritis. Arthritis and rheumatism. 2003;48(10):2741–9. Epub 2003/10/15. 10.1002/art.11223 . 14558078

[37] Ziegler AG, Rewers M, Simell O, Simell T, Lempainen J, Steck A, et al Seroconversion to multiple islet autoantibodies and risk of progression to diabetes in children. Jama. 2013;309(23):2473–9. Epub 2013/06/20. 10.1001/jama.2013.6285 . 23780460 PMC4878912

[38] Arbuckle MR, McClain MT, Rubertone MV, Scofield RH, Dennis GJ, James JA, et al Development of autoantibodies before the clinical onset of systemic lupus erythematosus. The New England journal of medicine. 2003;349(16):1526–33. Epub 2003/10/17. 10.1056/NEJMoa021933 . 14561795

[39] Ogawa Y, Sugiura K, Watanabe A, Kunimatsu M, Mishima M, Tomita Y, et al Autoantigenicity of DFS70 is restricted to the conformational epitope of C-terminal alpha-helical domain. Journal of autoimmunity. 2004;23(3):221–31. Epub 2004/10/27. 10.1016/j.jaut.2004.07.003 . 15501393

