# Supplementary figures and images for: Thrombophilia Associated with Anti-DFS70 Autoantibodies
Source: PLoS One. 2015 Sep 23;10(9):e0138671. doi: 10.1371/journal.pone.0138671 (PMC4580612; doi:10.1371/journal.pone.0138671)

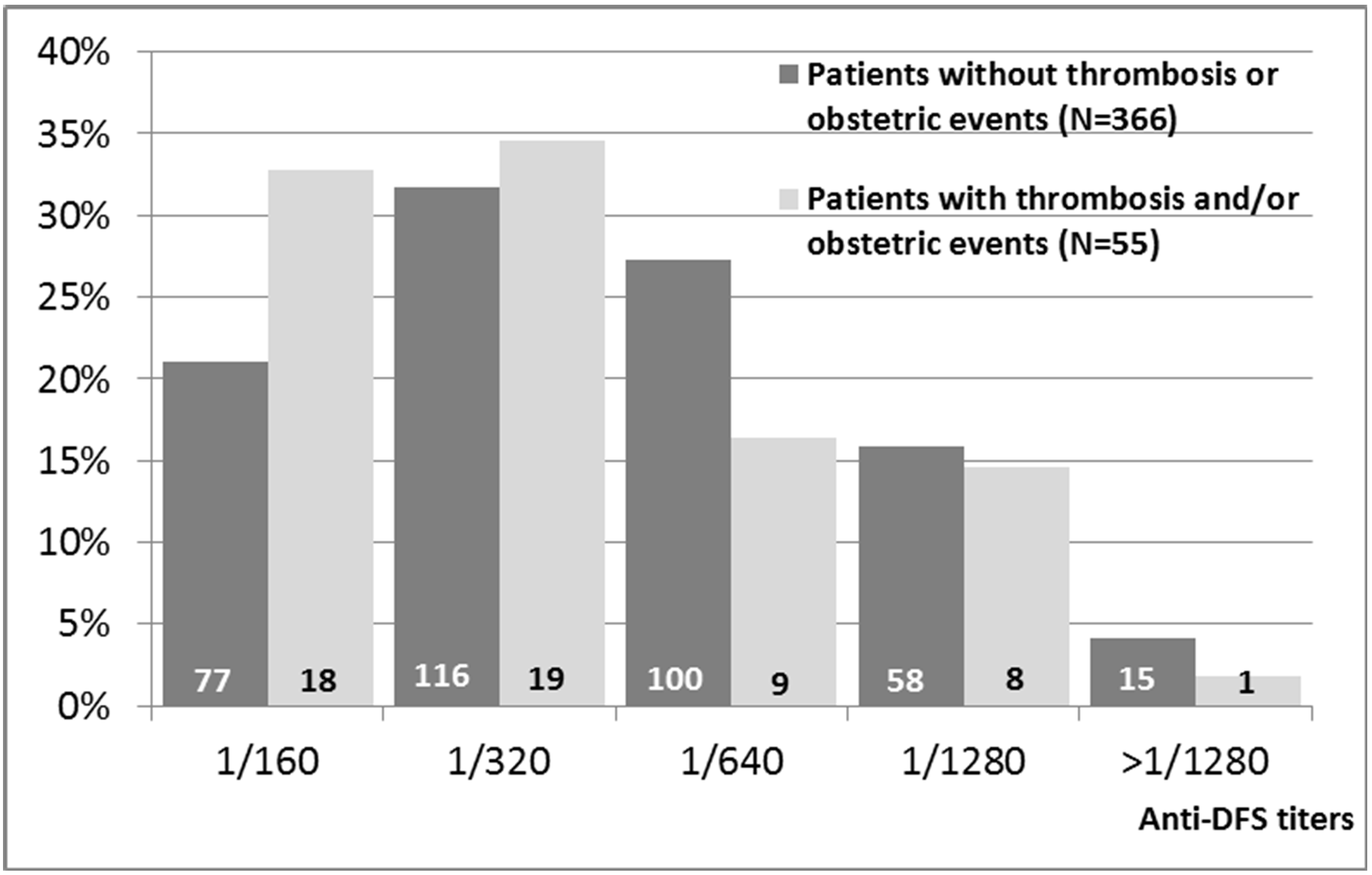

Supplement: S1 Fig — Distribution of anti-DFS70 antibodies titers in patients with or without thrombosis and/or obstetric complications is shown. (TIF) [file pone.0138671.s001.tif]
