# Supplementary material for: Thrombophilia Associated with Anti-DFS70 Autoantibodies
Source: PLoS One. 2015 Sep 23;10(9):e0138671. doi: 10.1371/journal.pone.0138671 (PMC4580612; doi:10.1371/journal.pone.0138671)
Supplement: S1 Table — F, Female; M, Male; CTD, Connective tissue disease; SLE, Systemic Lupus Erythematosus; SjS, Sjögren Syndrom; RA, Rheumatoid arthritis; AT, Arterial thrombosis; VT, Venous thrombosis; OE, Obstetric event including miscarriage, preeclampsia or HELLP syndrome; OCP, Oral Contraceptive Pills containing estrogen; APL, Anti-phospholipid Antibodies; Htz, Heterozygous, Hmz, Homozygous; NA, Not available; *, Patients also included in the thrombosis group. Cardiovascular risk factors included age (>50 for men and >60 for women), history of myocardial infarction in first-degree relatives (<55 for men, <65 for women), tobacco use, type 2 diabetes, HDL-cholesterol <0.4g/L. †, miscarriages before the 10th week of gestation; ‡, fetal death after the 10th week of gestation; #, premature birth with eclampsia before the 34th week of gestation (DOC) [file pone.0138671.s002.doc]

| Patient | | | |  | | First thrombosis/obstetrical event | | |  | Number of events | | |  | Risk factors | | | |  |
| --- | --- | --- | --- | --- | --- | --- | --- | --- | --- | --- | --- | --- | --- | --- | --- | --- | --- | --- |
| N° | Referring physician | Age | Gender |  | | Age | Nature | Context |  | AT | VT | OE |  | Cardio- | Blood clotting | CTD | Anti-DFS |  |
|  |  |  |  |  |  |  |  |  |  |  |  |  |  | vascular | disorder |  | titer |  |
| 1 | Hematology | 23 | F | | 18 | | VT | Spontaneous | | 0 | 2 | 0 |  | 1 | PS deficit | No | 640 |  |
| 2 | Hematology | 49 | F | | 59 | | VT | Immobilization | | 0 | 5 | 0 |  | 0 | PS deficit | No | 1280 |  |
| 3 | Hematology | 23 | M | | 34 | | VT | Spontaneous | | 0 | 2 | 0 |  | 0 | 0 | No | 160 |  |
| 4 | Hematology | 36 | F | | 30 | | AT | OCP | | 2 | 0 | 0 |  | 1 | 0 | No | 320 |  |
| 5 | Hematology | 17 | F | | 16 | | VT | OCP | | 0 | 1 | 0 |  | 0 | 0 | No | 160 |  |
| 6 | Hematology | 43 | F | | 32 | | VT | OCP & | | 0 | 2 | 0 |  | 0 | 0 | No | 1280 |  |
|  |  |  |  |  |  |  |  | Immobilization | |  |  |  |  |  |  |  |  |  |
| 7* | Hematology | 34 | F | | 21 | | VT | NA | | 0 | 2 | 0 |  | 0 | APL | No | 160 |  |
| 8* | Hematology | 70 | F | | 32 | | VT | Pregnancy | | 0 | 3 | 0 |  | 1 | 0 | No | 160 |  |
| 9* | Hematology | 45 | F | | 31 | | VT | Pregnancy | | 0 | 2 | 0 |  | 0 | 0 | No | 320 |  |
| 10* | Hematology | 21 | M | | 19 | | VT | Spontaneous | | 0 | 1 | 0 |  | 1 | 0 | No | 160 |  |
| 11 | Internal Medicine | 29 | M | | 22 | | VT | Plane flight | | 0 | 1 | 0 |  | 0 | NA | No | 640 |  |
| 12 | Internal Medicine | 37 | F | | 33 | | OE | Spontaneous | | 0 | 0 | 1 |  | NA | NA | No | 1280 |  |
| 13 | Internal Medicine | 46 | F | | 43 | | VT | Spontaneous | | 0 | 1 | 0 |  | 0 | 0 | No | 640 |  |
| 14 | Internal Medicine | 64 | F | | 48 | | VT | Spontaneous | | 0 | 5 | 0 |  | 1 | NA | No | 640 |  |
| 15 | Internal Medicine | 49 | F | | 38 | | OE | Spontaneous | | 0 | 0 | 3 |  | 0 | APL | SLE | 320 |  |
| 16 | Internal Medicine | 48 | F | | 23 | | VT | Spontaneous | | 0 | 2 | 0 |  | 1 | APL | No | 320 |  |
| 17 | Internal Medicine | 36 | F | | 26 | | OE | Spontaneous | | 0 | 0 | 3 |  | NA | 0 | No | 160 |  |
| 18 | Internal Medicine | 28 | F | | 25 | | OE | Spontaneous | | 0 | 0 | 3 |  | 0 | 0 | No | 1280 |  |
| 19 | Internal Medicine | 39 | F | | 37 | | AT | Spontaneous | | 1 | 0 | 0 |  | 0 | PS | SLE | 640 |  |
| 20 | Internal Medicine | 23 | M | | 19 | | AT | Spontaneous | | 1 | 0 | 0 |  | 2 | V Leiden (Htz)  MTHFR C677T (Hmz) | No | 1280 |  |
| 21 | Internal Medicine | 66 | M | | 63 | | VT | Spontaneous | | 0 | 2 | 0 |  | 4 | MTHFR C677T (Hmz) | No | 320 |  |
| 22 | Internal Medicine | 38 | F | | 31 | | OE | Spontaneous | | 0 | 0 | 3 |  | NA | 0 | No | 1280 |  |
| 23 | Hematology | 33 | F | | 29 | | VT | Spontaneous | | 0 | 2 | 0 |  | 1 | 0 | SLE | 320 |  |
| 24 | Infectious Diseases | 58 | M | | 45 | | AT | Spontaneous | | 1 | 0 | 0 |  | 4 | NA | No | 160 |  |
| 25 | Internal Medicine | 45 | F | | 43 | | VT | NA | | 0 | >1 | 1 |  | NA | APL | SLE | 320 |  |
| 26 | Internal Medicine | 57 | F | | 38 | | VT | OCP | | 0 | 4 | 0 |  | 0 | 0 | No | 320 |  |
| 27 | Rhumatology | 75 | F | | 44 | | OE | Spontaneous | | 0 | 0 | 3 |  | 3 | NA | RA | 160 |  |
| 28 | Internal Medicine | 36 | F | | 24 | | OE | Spontaneous | | 0 | 0 | >6 |  | 0 | 0 | No | 640 |  |
| 29 | Internal Medicine | 53 | F | | 38 | | OE | Spontaneous | | 0 | 0 | 1 |  | NA | NA | No | 160 |  |
| 30 | Internal Medicine | 50 | F | | 49 | | AT | NA | | 0 | 1 | 0 |  | NA | 0 | No | 1280 |  |
| 31 | Visceral surgery | 28 | F | | 25 | | OE | Spontaneous | | 0 | 1 | 2 |  | 0 | NA | No | 320 |  |
| 32 | Internal Medicine | 54 | F | | 37 | | OE | Spontaneous | | 0 | 0 | 3 |  | 1 | NA | No | 160 |  |
| 33 | Internal Medicine | 37 | F | | 24 | | VT | OCP | | 0 | 1 | 3 |  | 1 | APL | No | 160 |  |
| 34 | Internal Medicine | 61 | F | | 56 | | AT | Spontaneous | | 1 | 0 | 0 |  | 1 | 0 | No | 1280 |  |
| 35 | Internal Medicine | 48 | F | | 38 | | AT | Spontaneous | | 1 | 0 | 2 |  | 1 | NA | No | 320 |  |
| 36 | Internal Medicine | 37 | F | | 36 | | OE | Spontaneous | | 0 | 0 | 3 |  | 1 | NA | SLE | 160 |  |
| 37 | Internal Medicine | 75 | M | | 50 | | AT | Spontaneous | | 4 | 0 | 0 |  | 3 | MTHFR C677T (Hmz) | No | 160 |  |
| 38 | Internal Medicine | 54 | F | | 39 | | OE | Spontaneous | | 0 | 0 | 3 |  | 0 | NA | SjS | 640 |  |
| 39 | Internal Medicine | 29 | F | | 24 | | OE | Spontaneous | | 0 | 0 | 2 |  | 0 | NA | No | 160 |  |
| 40 | Internal Medicine | 83 | M | | NA | | AT | NA | | 1 | 0 | 0 |  | 1 | NA | No | 320 |  |
| 41 | Internal Medicine | 58 | F | | NA | | OE | Amniocentesis | | 0 | 0 | 1 |  | 0 | NA | No | 640 |  |
| 42 | Internal Medicine | 25 | F | | 25 | | AT | OCP+Cardiopathy | | 1 | 0 | 0 |  | 0 | 0 | No | 320 |  |
| 43 | Internal Medicine | 30 | F | | 18 | | VT | Spontaneous | | 0 | 1 | 0 |  | 0 | APL | SLE | 640 |  |
| 44 | Internal Medicine | 67 | F | | 52 | | AT | Spontaneous | | 1 | 0 | 0 |  | 5 | NA | No | 320 |  |
| 45 | Internal Medicine | 59 | M | | 58 | | AT | Spontaneous | | 1 | 0 | 0 |  | 1 | 0 | No | 1280 |  |
| 46 | Internal Medicine | 48 | F | | 47 | | AT | Spontaneous | | 3 | 0 | 0 |  | 1 | 0 | No | 640 |  |
| 47 | Internal Medicine | 30 | F | | 30 | | OE | Spontaneous | | 0 | 0 | 7 |  | 0 | APL | No | 160 |  |
| 48 | Internal Medicine | 25 | F | | 25 | | VT | Immobilisation | | 0 | 1 | 0 |  | 0 | NA | No | 1280 |  |
| 49 | Internal Medicine | 33 | F | | 28 | | OE | Spontaneous | | 0 | 0 | 1 |  | 1 | APL | No | 320 |  |
| 50 | Internal Medicine | 25 | F | | 24 | | VT | Spontaneous | | 0 | 1 | 0 |  | 1 | NA | No | 160 |  |
| 51 | Internal Medicine | 44 | F | | 32 | | VT | NA | | 0 | 1 | 0 |  | NA | NA | SjS | 1280 |  |
| 52 | Hematology | 74 | M | | NA | | VT | NA | | 0 | 2 | 0 |  | 1 | NA | No | 320 |  |
| 53 | Internal Medicine | 50 | M | | 50 | | TA | Spontaneous | | 1 | 0 | 0 |  | 1 | NA | No | 640 |  |
| 54 | Internal Medicine | 43 | F | | 38 | | TO | Spontaneous | | 0 | 0 | 1 |  | 1 | APL | No | 160 |  |
| 55* | Hematology | 28 | F | | NA | | VT | Spontaneous | | 0 | 1 | 0 |  | NA | NA | No | 320 |  |
